# A Rare Presentation of Coronavirus Disease 2019 (COVID-19) Induced Viral Myositis With Subsequent Rhabdomyolysis

**DOI:** 10.7759/cureus.8074

**Published:** 2020-05-12

**Authors:** Qian Zhang, Khine S Shan, Artem Minalyan, Conor O'Sullivan, Travis Nace

**Affiliations:** 1 Internal Medicine, Abington Hospital-Jefferson Health, Abington, USA; 2 Internal Medicine, University of Maryland Medical Center, Baltimore, USA; 3 Library Science, Abington Hospital-Jefferson Health, Abington, USA

**Keywords:** covid-19, novel coronavirus, coronavirus, viral myositis, myositis, rhabdomyolysis

## Abstract

A 38-year-old gentleman with no significant past medical history but had recent COVID-19 exposure presented to the hospital with the chief complaints of fever, shortness of breath, and generalized myalgia. He was unfortunately found to be severe acute respiratory syndrome coronavirus 2 (SARS-CoV-2) positive. Laboratory findings showed creatine kinase (CK) >42,670 U/L along with elevated inflammatory markers and unremarkable creatinine, cardiac troponin level. The cause of his rhabdomyolysis was discovered to be due to COVID-19 as he had no evidence of other viral infections, strenuous exercise, seizure, or other nontraumatic exertional etiologies. He received aggressive fluid resuscitation while we trended his CK levels along with other inflammatory markers throughout his hospitalization course. His diffuse myalgia improved with treatments, and he was found to maintain stable hemodynamics and was subsequently discharged home.

## Introduction

Novel coronavirus disease 2019 or COVID-19 is an emerging viral infection that originated from Wuhan, China. It had quickly spread across the globe within a few months with significant morbidity and mortality. It is caused by a novel enveloped single-stranded RNA betacoronavirus which is known as the severe acute respiratory syndrome coronavirus 2 (SARS-CoV-2). Typical symptoms include fever, cough, dyspnea, fatigue, and myalgia [1]. It rarely can cause headaches, diarrhea, and hemoptysis [2]. However, patients may present with atypical symptoms that make their diagnosis challenging [3]. We present a case of a 38-year-old man who initially presented with high fever, dyspnea, and severe myalgia but was discovered to have acute viral myositis complicated by rhabdomyolysis due to SARS-CoV-2. 

## Case presentation

The patient is a 38-year-old gentleman presented with the chief complaint of fever and dyspnea. His past medical history was significant for sleep apnea and gastroesophageal reflux. He denied any recent travel history. He smoked cigarettes occasionally, but denied illicit drug use. He worked as a postmaster and was exposed to a co-worker with COVID-19 infection. He started to develop a cough with occasional sputum production a few days later. He also had severe myalgia and back pain. He decided to seek medical attention as his symptoms persisted for another few days without resolution. 

**Figure 1:**
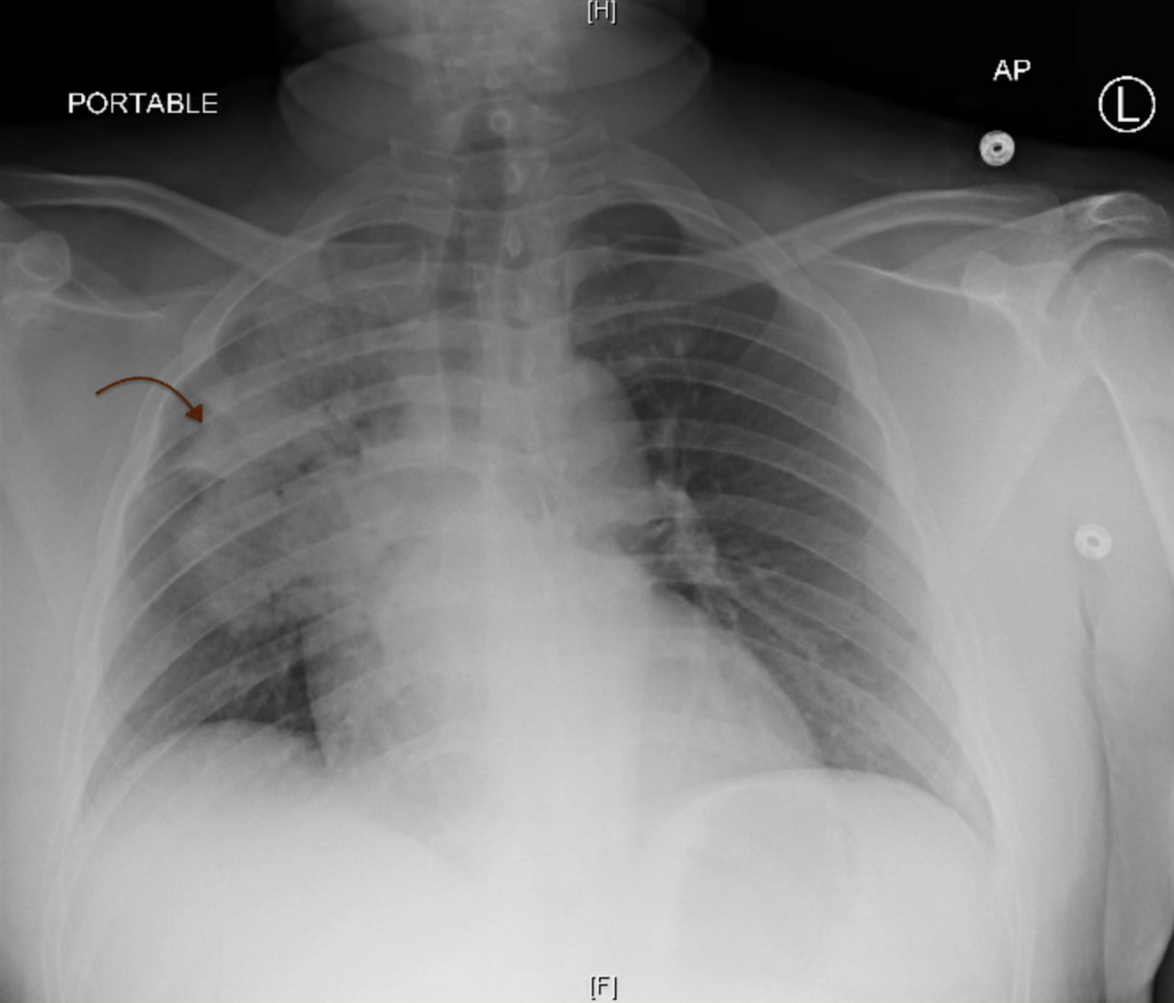
Chest X-Ray Consolidation found in the right upper and middle lobes with the presence of air bronchograms. H, head; F, foot, AP, anterior/posterior view.

In the emergency department (ED), his initial vital signs included temperature 104.8°F, blood pressure 136/81 mmHg, respiratory rate 20 breaths per minute, heart rate 120 beats per minute, and oxygen saturation 95% on room air. Physical examination revealed a well-developed, a well-nourished man appeared to be lethargic, diaphoretic, with bilateral diminished breath sounds and had intact motor and sensation throughout the body. Pertinent laboratory findings included the following: aspartate aminotransaminase (AST) 492 U/L, alanine aminotransaminase (ALT) 110 U/L, creatine kinase (CK) >42,670 U/L, creatinine 0.98 mg/dL, white blood count (WBC) 12.2 K/UL, absolute lymphocyte count 1.1 K/UL, C-reactive protein (CRP) 364 mg/L, lactate dehydrogenase (LDH) 4,301 U/L, and positive SARS-COV-2. Cardiac troponin, blood cultures, coagulation panel, influenza A/B target RNA, and respiratory syncytial virus (RSV) target RNA were unremarkable. Chest x-ray showed right upper and middle lobe consolidation with an air bronchogram that was concerning for multifocal pneumonia (Figure 1). He was placed on continuous pulse oximetry and began empirical treatment with cefepime and azithromycin. He was also started on continuous fluid resuscitation due to concerns of rhabdomyolysis with extremely elevated CK levels despite no evidence of strenuous exercise, seizure, or other nontraumatic exertional causes. Electrocardiography (EKG) was unremarkable. He was placed on airborne precautions and remained to be clinically stable overnight. 

**Figure 2:**
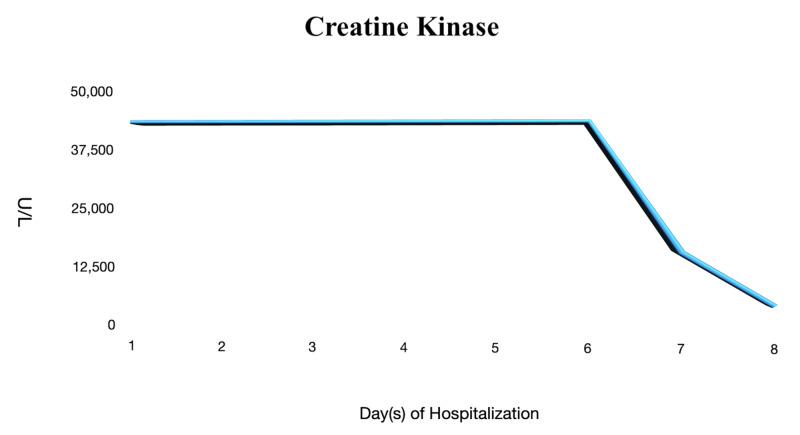
Creatine Kinase The graph shows persistent elevation of creatine kinase (CK) >42,670 U/L for six days prior to a decrease in the levels.

On days 2 and 3 of hospitalization, he remained to be febrile with the temperature max (Tmax) of 102.6°F while breathing comfortably on ambient air. He was placed on two liters of nasal cannula temporarily. He still complained of generalized myalgia and whole-body muscle soreness. Furthermore, his serum biomarkers were trended on a daily basis as they continued to elevate. There were concerns about SARS-CoV-2 viral myositis given his extremely elevated CK levels (Figure 2). He was fluid resuscitated continuously despite negative urine myoglobin. Moreover, he was treated with hydroxychloroquine in the setting of a normal QTc interval found on EKG. It was decided to substitute azithromycin with doxycycline to avoid the additive effects of azithromycin and hydroxychloroquine on the QTc interval.

**Figure 3:**
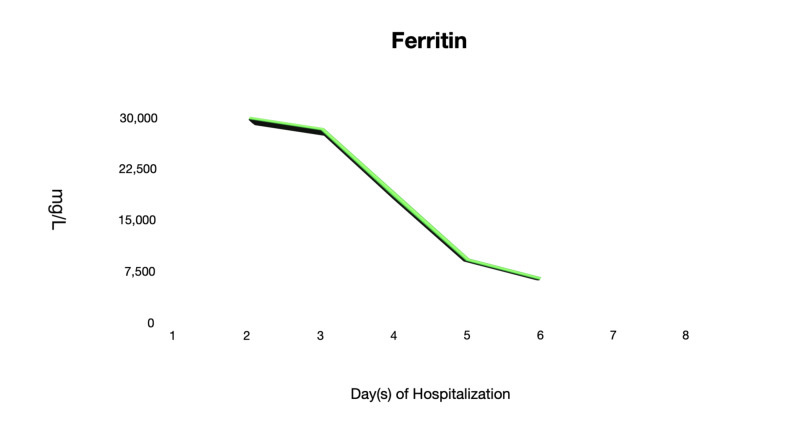
Ferritin The ferritin levels throughout the hospitalization course.

**Figure 4:**
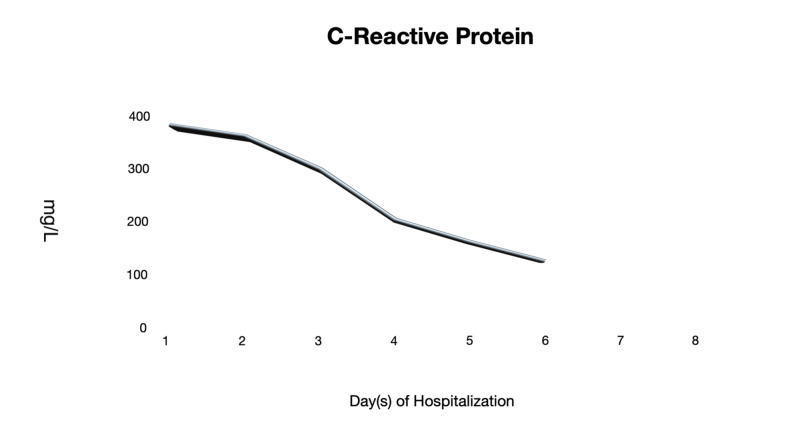
C-Reactive Protein (CRP) The CRP levels throughout the hospitalization course.

**Figure 5:**
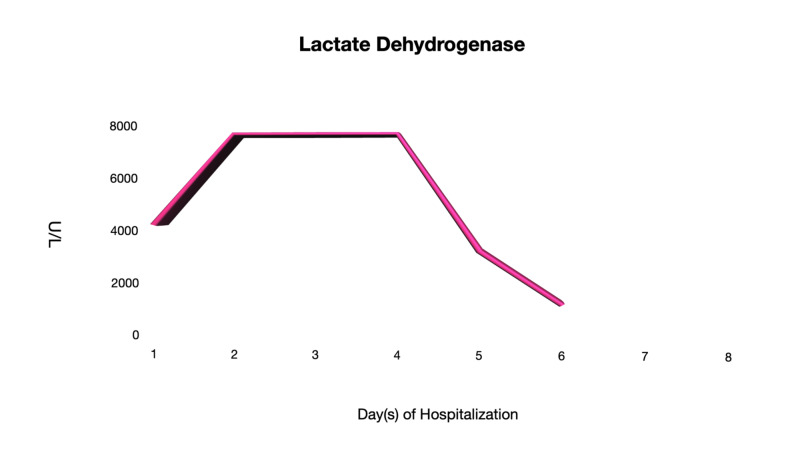
Lactate Dehydrogenase (LDH) The LDH levels throughout the hospitalization course.

On days 4 and 6 of hospitalization, he remained to be clinically stable while on room air. He remained to be afebrile and was able to ambulate in the room without dyspnea. His generalized myalgia improved despite that CK levels remained to be elevated. Other inflammatory markers such as LDH, CRP, and ferritin levels were trending downwards (Figures 3-5). Antibiotics were discontinued as there were low clinical suspicions of superimposed bacterial pneumonia. 

On days 7 and 8 of hospitalization, CK levels began to trend downwards as his generalized myalgia remained to be in remission. He was afebrile and denied to be in respiratory distress. He continued to receive fluid resuscitation and was subsequently discharged the following day. He was advised to remain hydrated after discharge and follow-up with his primary care physician to further monitor his CK levels.

## Discussion

Diffuse myalgias may occur frequently during the prodromal phase of any acute viral infection. However, acute viral myositis rarely occurs in the adult population even though it is common in the pediatric population. Myositis may present with a higher intensity of pain than that of generalized myalgia [4]. Viral myositis is often self-limited, lasting on an average of three days but can be complicated by rhabdomyolysis [5]. Clinicians should be aware of myositis-induced rhabdomyolysis when patients report myalgia and weakness despite they are common symptoms of a viral infection.

Various viruses may cause myositis or virus-induced rhabdomyolysis. Viral myositis and rhabdomyolysis are most commonly caused by influenza A and B, with enteroviruses, human immunodeficiency virus, cytomegalovirus, Epstein-Barr virus, herpes simplex virus, and varicella-zoster virus being less common. Approximately 3% of patients with influenza-induced myositis developed rhabdomyolysis per literature review of 300 cases [4]. Laboratory results usually show elevated CK, LDH, and AST levels. There may be myopathic changes on the electromyogram. However, the diagnosis of myositis does not require muscle biopsy but rather is diagnosed clinically in the setting of viral infection [4,6]. Influenza A/B target RNA was checked during admission for our patient with subsequent negative results that narrowed down the cause of rhabdomyolysis to be likely secondary to COVID-19 viral myositis. Treatment is usually supportive, as myositis is a self-limited condition. However, it may rarely proceed to rhabdomyolysis and acute kidney failure [5]. 

Predisposing factors for rhabdomyolysis include infection, crush injury, electrolyte abnormalities, substance abuse, alcohol use, mechanical fall, drugs, or autoimmune myopathies [7]. Jin and Tong recently reported a 60-year-old man from Wuhan, China with SARS-CoV-2, who was found to have rhabdomyolysis on day 9 of hospitalization when he developed pain and weakness of his lower extremities [8]. However, another case report described an 88-year-old man who initially presented with rhabdomyolysis with bilateral thigh weakness and pain later found to have SARS-CoV-2 [9]. In both case reports, the CK level was below 13,000 U/L, unlike our patient who presented with a CK level >42,670 U/L. Moreover, both cases emphasized the fact that rhabdomyolysis could be either an initial presentation or a late complication [8,9]. Patients with COVID-19 are at risk for worsening respiratory distress from fluid overload as it is also important to prevent acute kidney injury at the same time [9]. This was reflected in a case reported by Suwanwongse and Shabarek when the patient received small boluses of 250 ml intravenous fluid with the close clinical observation while trended the CPK levels [9]. Chen et al. previously described three cases of SARS-CoV associated with viral myositis complicated by rhabdomyolysis [7]. 

There are several proposed mechanisms of action of viral-induced myositis despite the lack of in-depth research. Cases reported that direct viral invasion to muscles may damage myocytes with subsequent rhabdomyolysis development [10]. Children are more susceptible to viral myositis due to virus tropism toward more immature muscles as seen in animal studies [4]. Moreover, an immune-mediated process can be explained by four possible mechanisms: deposition of virus-antibody complexes in muscles with an “innocent bystander” damage; immunologic cross-reactivity as a result of homology between viral antigens and human muscle proteins; transformation of antigens on muscle membrane by adsorption of viral particles; and virus-induced expression of antigens on the cell membrane [4,10]. Lastly, circulating viral toxins directly attacking the myocyte membranes could damage muscle fibers [11]. Some evidence of T-cell-mediated injury was shown on muscle biopsy in some cases of patients with viral myositis [6].

Our patient did not develop acute kidney failure as a complication of rhabdomyolysis was likely due to early recognition of rhabdomyolysis, and thus began prompt treatment with aggressive intravenous fluids. In addition, it is important to consider rhabdomyolysis and monitor CK levels if the patient presented with myalgia with concerns of viral infection. In a study by Guan et al., 13.7% of patients with COVID-19 had CK levels above 200 U/L, but the CK levels did not correlate with the disease severity [1]. There are currently a few published case reports of rhabdomyolysis associated with the new SAR-CoV-2 even though previous SAR-CoV rarely had caused rhabdomyolysis. Our patient is the first reported case report with impressive CK levels without kidney failure with concurrent muscle weakness. 

## Conclusions

Novel COVID-19 is one of the most discussed topics throughout the world as it has impacted the daily life of every individual across the globe. Clinical suspicion of viral myositis-induced rhabdomyolysis should be a part of the clinical differential diagnosis if a patient presents with diffuse body myalgia along with a positive SAR-CoV-2 result. Aggressive fluid resuscitation and daily CK level trending are important to treat and monitor the clinical progression as well as the response to treatment.
